# Identification of bifurcation transitions in biological regulatory networks using Answer-Set Programming

**DOI:** 10.1186/s13015-017-0110-3

**Published:** 2017-07-20

**Authors:** Louis Fippo Fitime, Olivier Roux, Carito Guziolowski, Loïc Paulevé

**Affiliations:** 1 LS2N, UMR CNRS 6004, Ecole Centrale de Nantes, Nantes, France; 2 LRI UMR 8623, Univ. Paris-Sud-CNRS, Université Paris-Saclay, 91405 Orsay, France; 3 Laboratoire d’Informatique de Paris Nord (LIPN), UMR 7030, Université Paris 13, 99 avenue Jean-Baptiste Clément, 93430 Villetaneuse, France

## Abstract

**Background:**

Numerous cellular differentiation processes can be captured using discrete qualitative models of biological regulatory networks. These models describe the temporal evolution of the state of the network subject to different competing transitions, potentially leading the system to different attractors. This paper focusses on the formal identification of states and transitions that are crucial for preserving or pre-empting the reachability of a given behaviour.

**Methods:**

In the context of non-deterministic automata networks, we propose a static identification of so-called bifurcations, i.e., transitions after which a given goal is no longer reachable. Such transitions are naturally good candidates for controlling the occurrence of the goal, notably by modulating their propensity. Our method combines Answer-Set Programming with static analysis of reachability properties to provide an under-approximation of all the existing bifurcations.

**Results:**

We illustrate our discrete bifurcation analysis on several models of biological systems, for which we identify transitions which impact the reachability of given long-term behaviour. In particular, we apply our implementation on a regulatory network among hundreds of biological species, supporting the scalability of our approach.

**Conclusions:**

Our method allows a formal and scalable identification of transitions which are responsible for the lost of capability to reach a given state. It can be applied to any asynchronous automata networks, which encompass Boolean and multi-valued models. An implementation is provided as part of the Pint software, available at http://loicpauleve.name/pint.

**Electronic supplementary material:**

The online version of this article (doi:10.1186/s13015-017-0110-3) contains supplementary material, which is available to authorized users.

## Introduction

The emerging complexity of dynamics of biological networks, and in particular of signalling and gene regulatory networks, is mainly driven by the interactions between the species, and the numerous feedback circuits they generate [1–4]. One of the prominent and fascinating features of cells is their capability to differentiate: starting from a multi-potent state (for instance, a stem cell), cellular processes progressively confine the cell dynamics in a narrow state space, an attractor. Deciphering those decision processes is a tremendous challenge, with important applications in cell reprogramming and regenerative medicine.

Qualitative discrete models of network dynamics, such as Boolean and multi-valued networks [5, 6], have been designed with such an ambition. These frameworks model nodes of the network by variables with small discrete domains, typically Boolean. Their value changes over time according to the state of their parent nodes. Exploring the dynamical properties of those computational models, such as reachability, i.e., the ability to evolve to a particular state, or attractors, i.e., the long-run behaviours, allows understanding part of important cellular processes [7–9].

Differentiation processes can be seen as processes making irreversible choices between nodes (genes) activations/inhibitions impacting the long term capabilities of the cell. For example, from a muti-potent state S, if a cell can later differentiate in two different types A and B, once in a type B, it can no longer change to type A without external perturbations. From a discrete dynamics perspective, those choices are modelled by transitions which make the system evolve from a multi-potent state where both A and B are possible in the future to a state where A is no longer reachable. Such decisive transitions, that we refer to as *bifurcation transitions*, are highly relevant to understand which entities and interactions play a key role during the cellular dynamics. Following this perspective, it is worth remarking that in the state where such a transition can occur, another transition exists which preserves the capability to reach A. Otherwise the decision that A is not reachable must have already been made previously.

**Fig. 1:**
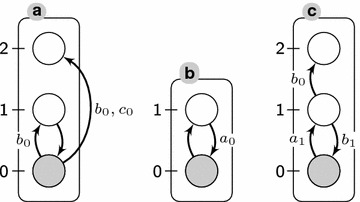
An example of automata network (AN). Automata are represented by *labelled boxes*, and local states by *circles* where ticks are their identifier within the automaton—for instance, the local state $a_0$ is the *circle* ticked 0 in the *box* *a*. A transition is a directed edge between two local states within the same automaton. It can be labelled with a set of local states of other automata. *Grayed* local states stand for the global state $\langle a_0, b_0, c_0 \rangle$

**Fig. 2:**
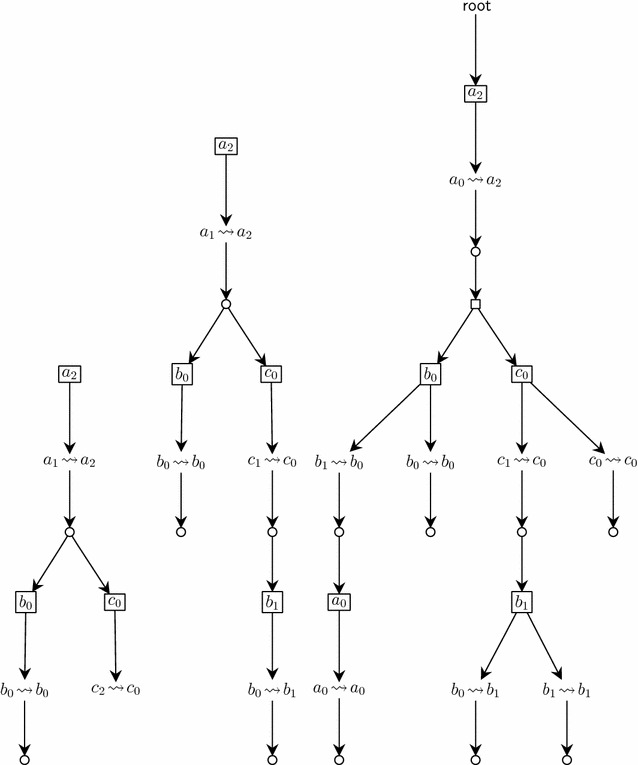
Examples of local causality graphs. (*Left*) over-approximation of $a_2$ reachability from $\langle a_1,b_0,c_2 \rangle$ (*middle*) over-approximation of $a_2$ reachability from $\langle a_1,b_0,c_1 \rangle$ (*right*) under-approximation of $a_2$ reachability from $\langle a_0,b_1,c_1 \rangle$. The *small circles* represent the local paths

Besides extracting precise knowledge on differentiation mechanisms in the discrete dynamics of the network, bifurcation transitions can *in fine* suggest drug targets for controlling cellular differentiation and/or counteracting pathological behaviours. Indeed, if it is ensured that the bifurcation is triggered in the appropriate state, then the reachability of a state of interest would be certainly prevented. On the other hand, blocking all bifurcation transitions in the appropriate states would ensure that the state of interest is inevitably reached.

In this article, we formally introduce the notion of bifurcation transitions in discrete dynamics of automata networks (ANs) and we provide a scalable method for their identification that relies on declarative programming with Answer-Set Programming (ASP) [10]. ANs allow encoding exactly the dynamics of asynchronous Boolean and multi-valued networks which are also known as Thomas networks [11]. We first show that bifurcation transitions can be completely identified using computation-tree temporal logic (CTL). However, this characterization relies extensively on the reachability problem, which is PSPACE-complete in ANs and similar frameworks [12], which limits its tractability. The main contribution of this paper is the introduction of an approximation of the bifurcation identification which is NP. In order to obtain an approach tractable on large biological networks, we show a combination of methods of static analysis of ANs dynamics [13, 14], concurrency theory, and constraint programming for relaxing efficiently the bifurcation problem. Our method identifies correct bifurcations only (no false positives) but, due to the embedded approximations, is incomplete (false negatives may exist). To our knowledge, this is the first integrated method to extract bifurcation transitions from discrete models of large interaction networks.

The output of our method is a set of transitions, for instance “activation of gene x by active genes y and z”, and optionally the set of states in which their occurrence removes the capability to reach the goal. It is worth noticing that bifurcation transitions are transitions of the input model which play a crucial role for the goal reachability. They do not directly provide targets for controlling the system. Therefore, bifurcation transitions are different from intervention sets [15, 16] or cut sets [17, 18] which propose perturbations to apply on a system in order to enforce/prevent the occurrence of a state/reaction of interest. Whereas these predictions can help to control the reachability of an attractor, they do not allow to directly understand the structure of the original model dynamics, notably how the different attraction basins are connected. Bifurcation transitions precisely indicate when and how the system exits a state where a capability was reachable.

## Background

### Automata networks

An AN is a finite set of finite-state machines that have transitions between their local states determined by the state of other automata in the network. The global state space of the network is the product of the local states of the individual automata. The local transitions specify the current and successor local state of an automaton, possibly constrained by the state of other automata.

#### **Definition 1**

An AN is defined by a tuple $(\Sigma ,S,T)$ where

- $\Sigma$ is the finite set of automata identifiers;
- For each $a\in \Sigma$, $S(a) = \{a_i,\dots ,a_j\}$ is the finite set of local states of automaton *a*; $S \mathop {=}\limits ^{\Delta }\prod _{a\in \Sigma } S(a)$ is the finite set of global states; $L\mathop {=}\limits ^{\Delta }\bigcup _{a\in \Sigma } S(a)$ denotes the set of all the local states.
- $T = \{ a \mapsto T_a \mid a\in \Sigma \}$, where $\forall a\in \Sigma , T_a \subseteq S(a)\times 2^{L\setminus S(a)} \times S(a)$ with $(a_i,\ell ,a_j)\in T_a \Rightarrow a_i\ne a_j$ and $\forall b\in \Sigma , |\ell \cap S(b)| \le 1$, is the mapping from automata to their finite set of local transitions.

We write $t=a_i\xrightarrow \ell a_j\in T \mathop {\Leftrightarrow }\limits ^{\Delta }(a_i,\ell ,a_j)\in T(a)$, and $\ell$ is referred to as the *enabling condition* of the transition *t*.

At any time, each automaton is in one and only one local state, which forms the global state of the network. Assuming an arbitrary ordering between automata identifiers, the set of global states of the network is referred to as $S$ as a shortcut for $\prod _{a\in \Sigma }S(a)$. Given a global state $s\in S$, $s({a})$ is the local state of automaton *a* in *s*, i.e., the *a*th coordinate of *s*.

**Fig. 3:**
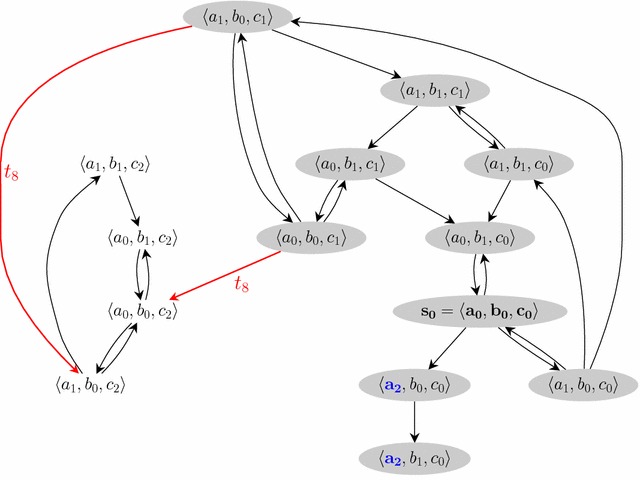
Transition graph of the AN in Fig. 1 from the initial state $s_0=\langle a_0,b_0,c_0 \rangle$ in *bold*. The goal $a_2$ is in *bold* and *blue*; the states connected to the goal are in *grey*; the bifurcations for the goal are in *thick/red* and are labelled with the name of the local transitions in the AN definition

**Fig. 4:**
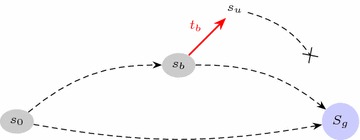
General illustration of a bifurcation. $s_0$ is the initial state, $S_g$ is a set of states in which the goal local state is present. The *dashed arrows* represent a sequence (possibly empty) of transitions. The *plain red arrow* is a bifurcation from a global state $s_b$ to $s_u$, and $t_b$ is the associated local transition

A local transition $t= {a}_{i} \xrightarrow {\ell } {a}_{j}\in T$ is applicable in a global state $s\in S$ when $a_i$ and all the local states in $\ell$ are in *s*. The application of the local transition, noted $s\cdot t$, replaces the local state of *a* with $a_j$ (Definition 2).

#### **Definition 2**

(*Transition, reachability*) Given a state $s\in S$ and a local transition $t= {a}_{i} \xrightarrow {\ell } {a}_{j}\in T$ such that $s({a})=a_i$ and $\forall b_k\in \ell$, $s({b})=b_k$, $s\cdot t$ is the state *s* where $a_i$ has been replaced by $a_j$:

$$\begin{aligned} \forall b\in \Sigma , (s\cdot t)({b}) = {\left\{ \begin{array}{ll} a_j & \quad \text {if}\; b=a\\ s(b) & \quad \text {otherwise} \end{array}\right. } \end{aligned}$$

We then write $s \xrightarrow t s'$ where $s'=s\cdot t$. The *reachability* binary relation $\rightarrow ^{*}~\subseteq S\times S$ satisfies

$$\begin{aligned} s \rightarrow ^{*}s' \mathop {\Leftrightarrow }\limits ^{\Delta }s=s' \vee \exists t\in T: s\xrightarrow t s'' \wedge s''\rightarrow ^{*}s' \end{aligned}$$

In this paper, we consider the *asynchronous* semantics of ANs: only one local transition can be applied at a time. In this asynchronous semantics, different local transitions may be applicable to the same state, each of them leading to different behaviours. The choice of the transition is *non-deterministic*. A global state $s'$ is reachable from *s*, noted $s\rightarrow ^{*}s'$, if and only if there exists a (possibly empty) sequence of transitions leading from *s* to $s'$. Finally, an attractor is a smallest set of states from which no transition can exit. They correspond to the long-term dynamics of the network:

#### **Definition 3**

(*Attractor*) An *attractor* of the AN $(\Sigma ,S,T)$ is a set of states $A\subseteq S$ such that

- *A* is *strongly connected* w.r.t. $\rightarrow ^{*}$: $\forall s,s'\in A, s\rightarrow ^{*}s'$; and
- *A* is *terminal* w.r.t. $$\rightarrow ^{*}$$: $$\forall s\in A$$, $$\exists s'\in S: s\rightarrow ^{*}s' \Rightarrow s'\in A$$.

Figure 1 represents an AN $(\Sigma ,S,T)$ of 3 automata ($\Sigma = \{a,b,c\}$), with $S(a) = \{a_0,a_1,a_2\}$, $S(b) = \{b_0,b_1\}$, $S(c) = \{c_0,c_1,c_2\}$, and 8 local transitions defined as follows:

$$\begin{aligned} T(a)&= \{t_1= {a}_{1} \xrightarrow {\emptyset } {a}_{0}, t_2= {a}_{0} \xrightarrow {b_0} {a}_{1}, t_3= {a}_{0} \xrightarrow {b_0,c_0} {a}_{2}\} \\ T(b)&= \{t_4= {b}_{0} \xrightarrow {\emptyset } {b}_{1}, t_5= {b}_{1} \xrightarrow {a_0} {b}_{0}\} \\ T(c)&= \{t_6= {c}_{0} \xrightarrow {a_1} {c}_{1}, t_7= {c}_{1} \xrightarrow {b_1} {c}_{0}, t_8= {c}_{1} \xrightarrow {b_0} {c}_{2}\} \end{aligned}$$

From the given initial state $s_0=\langle a_0,b_0,c_0 \rangle$, 3 transitions can be applied: $t_2$, $t_3$, and $t_4$; the application of the latter results in $s_0\cdot t_4 = \langle a_0,b_1,c_0 \rangle$ (automaton *b* is now in state $b_1$).

### Encoding Boolean and Thomas networks with automata networks

The asynchronous semantics of any Boolean network or Thomas (multi-valued) network can be encoded equivalently with ANs [11]. Note that, according to Thomas networks semantics, the transitions increment or decrement by one the level of node. Hence, ANs encoding Thomas networks have only transitions of the form ${a}_{i} \xrightarrow {\ell } {a}_{j}$ with $|i-j|=1$.

Tools such as BioLQM 1 provide automatic translations from standard model formats for Boolean/Thomas networks to ANs.

### Reachability and formal approximations

In this section, we give a brief overview of the basics of reachability checking, stressing the methods we use in this paper.

#### State graph and partial order reductions

Given two states $s,s'$ of an AN (or an equivalent Petri net), verifying $s\rightarrow ^{*}s'$ is a PSPACE-complete problem [12].

The common approach for reachability checking is to build the (finite) set of all the states reachable from *s* until finding $s'$, by exploring all the possible transitions. However, such a set can be rapidly intractable with large models. Techniques relying on symbolic representations, notably using binary decision diagrams (BDDs) and variants [19] can improve the scalability of this approach by several orders of magnitude [20].

Typically, numerous transitions in ANs are *concurrent*: their application is independent from each other. For instance, if $t_1$ and $t_2$ are concurrent in a state *s*, one can apply indifferently $s\cdot t_1\cdot t_2$ and $s\cdot t_2\cdot t_1$. Such features can be exploited to provide compact representations of the reachable states in a concurrent system, taking into account the partial order of transition applications. Unfoldings, and more precisely their complete finite prefixes [21], allow computing efficiently such compact representations.

**Table 1:** Experimental results for the identification of bifurcation transitions depending if $(\mathrm{I3})$ or $(\mathrm{I3}^\#)$ is used, compared to a exact model checking (MC) using NuSMV [20]

Automata network	|States|	Goal	MC (NuSMV)		With $(\mathrm{I3})$		With $(\mathrm{I3}^\#)$	
			$|t_b|$	Time (s)	$|t_b|$	Time (s)	$|t_b|$	Time (s)
Lambda phage $|\Sigma |=4\quad |T|=11$	14	$\mathrm {CI}_2$	10	0.1	6	0.1	0	0.2
		$\mathrm {Cro}_2$	3	0.1	3	0.1	2	0.3
EGF/TNF $|\Sigma |=28\quad |T|=55$	3698	$\mathrm {NFkB}_0$	5	0.2	4	0.1	2	0.1
		$\mathrm {IKB}_1$	5	0.2	3	0.1	2	0.1
Th_th1 $|\Sigma |=101\quad |T|=381$	≈3.10^11^	$\mathrm {BCL6}_1$	8	13	6	16	5	23
		$\mathrm {TBET}_1$	11	14	5	10	4	24
Th_th2 $|\Sigma |=101\quad |T|=381$	≈10^12^	$\mathrm {GATA3}_1$	9	108	8	24	7	20
		$\mathrm {BCL6}_1$	7	570	5	25	4	25
Th_pluri $|\Sigma |=101\quad |T|=381$	>5.10^14^	BCL6_1_ IL21_1_ FOXP3_1_ TGFB_1_	Out-of-time		Out-of-time		2	32
							0	26
							4	56
							5	96

Models Th_th1, Th_th2, and Th_pluri are the same AN but have different initial states. $|\Sigma |$ is the number of automata, and $|T|$ the number of transitions; |states| is the number of reachable state from the initial state; in the case of Th_pluri, it is only a lower bound as we were not able to compute the full state space. $|t_b|$ is the number of identified bifurcation transitions (among $T$). Computation times have been obtained on an Intel® Core™ i7-4770 3.40GHz CPU with 16GiB of RAM

In this paper, one of our methods uses complete finite prefixes of unfoldings to compute the states that are reachable from a given initial state. Indeed, because biological networks are typically very large, but also very sparse (each node/automaton interacts with a few others, compared to the size of the network), they exhibit a high degree of concurrency for their transitions, making unfolding approaches very effective in practice.

#### Formal approximations

When facing a large AN, it may turn out that the reachable state space is too large for the aforementioned exact verification of reachability. Moreover, the complexity of the reachability problem can be prohibitive when numerous verifications have to be done, for instance when enumerating candidate initial states.

In this paper, we rely on the reachability approximations for ANs introduced in [13, 14]. We use both *over-approximations* (OA) and *under-approximations* (UA) of the reachability problem: $s\rightarrow ^{*}s'$ is true only if $\mathrm{OA}(s\rightarrow ^{*}s')$ is true and $s\rightarrow ^{*}s'$ is true if $\mathrm{UA}(s\rightarrow ^{*}s')$ is true; but the converses do not hold in general:

$$\begin{aligned} \mathrm{UA}(s\rightarrow ^{*}s')&\Rightarrow s\rightarrow ^{*}s' \Rightarrow \mathrm{OA}(s\rightarrow ^{*}s') \end{aligned}$$

The approximations rely on static analysis by abstract interpretation of AN dynamics. We give here the basic explanations for the over- and under-approximations. The analyses rely on the causal decomposition of the transitions in compositing automata, and result in necessary or sufficient conditions for a reachability property of the form $s\rightarrow ^{*}s'$.

The core objects are the *objectives* and their *local paths* within two local states $a_i$, $a_j$ of a same automaton *a*. We call ${{a}_{i}}\!\leadsto \!{{a}_{j}}$ an *objective* and define $\mathrm{local}\text{-}\mathrm{paths}({{a}_{i}}\!\leadsto \!{{a}_{j}})$ the set of the acyclic paths of local transitions between $a_i$ and $a_j$. Definition 4 gives the formalization of $\mathrm{local}\text{-}\mathrm{paths}$ where we use the following notations. Given a local transition $t= {a}_{i} \xrightarrow {\ell } {a}_{j}\in T$, $\mathrm{orig}(t)\mathop {=}\limits ^{\Delta }a_i$, $\mathrm{dest}(t)\mathop {=}\limits ^{\Delta }a_j$, $\mathrm{enab}(t)\mathop {=}\limits ^{\Delta }\ell$. Given $z\in \mathbb N$, ${\tau }=({\tau }^n)_{n=1,\dots ,z}$ is a *sequence* of local transitions indexed by $n\in \{1,\dots ,z\}$; $|{\tau }|=z$ is the length of the sequence ${\tau }$; and $\varepsilon$ denotes the empty sequence ($|\varepsilon |=0$).

##### **Definition 4**

(*Local-paths*) Given an objective ${{a}_{i}}\!\leadsto \!{{a}_{j}}$,

- If $$i=j$$, $$\mathrm{local}\text{-}\mathrm{paths}({{a}_{i}}\!\leadsto \!{{a}_{i}})\mathop {=}\limits ^{\Delta }\{\varepsilon \}$$;
- If $i\ne j$, a sequence ${\tau }$ of transitions in *T*(*a*) is in $\mathrm{local}\text{-}\mathrm{paths}({{a}_{i}}\!\leadsto \!{{a}_{j}})$ if and only if it satisfies the following properties:
  - $$\mathrm{orig}({\tau }^1) = a_i$$, $$\mathrm{dest}({\tau }^{|{\tau }|})=a_j$$,
  - $$\forall n, 1\le n< |{\tau }|$$, $$\mathrm{dest}({\tau }^n) = \mathrm{orig}({\tau }^{n+1})$$,
  - $$\forall n,m, |{\tau }|\ge n > m\ge 1, \mathrm{dest}({\tau }^n)\ne \mathrm{orig}({\tau }^m)$$.

We write $t\in {\tau }\mathop {\Leftrightarrow }\limits ^{\Delta }\exists n, 1\le n \le |{\tau }|: {\tau }_n=t$. Given a local path ${\tau }$, ${\mathrm{enab}({\tau })}$ denotes the union of the conditions of all the local transitions composing it:

$$\begin{aligned} \textstyle {\mathrm{enab}({\tau })}\mathop {=}\limits ^{\Delta }\bigcup _{n=1}^{|{\tau }|} \mathrm{enab}({\tau }^n) \end{aligned}$$

In the AN of Fig. 1, $\mathrm{local}\text{-}\mathrm{paths}({{a}_{0}}\!\leadsto \!{{a}_{2}}) = \{ ( {a}_{0} \xrightarrow {b_0,c_0} {a}_{2})\}$; $\mathrm{local}\text{-}\mathrm{paths}({{c}_{0}}\!\leadsto \!{{c}_{2}})=\{ ( {c}_{0} \xrightarrow {a_1} {c}_{1}, {c}_{1} \xrightarrow {b_0} {c}_{2}) \}$; $\mathrm{local}\text{-}\mathrm{paths}({{c}_{2}}\!\leadsto \!{{c}_{1}})=\emptyset$.

Focusing on the reachability of a single local state $g_1$ from a state *s* where $s({g})=g_0$, the analyses essentially start with the local paths in $\mathrm{local}\text{-}\mathrm{paths}({{g}_{0}}\!\leadsto \!{{g}_{1}})$: if $g_1$ is reachable, then at least one of the local paths ${\tau }$ has to be realizable, meaning that all the local states of its conditions (${\mathrm{enab}({\tau })}$) should be reachable. This leads to a recursive reasoning by repeating the procedure with the objectives from *s* to the local states in ${\mathrm{enab}({\tau })}$.

The dependence relationships between the local paths of the different automata can be represented as a graph, where the nodes are all the local states, all the possible objectives, and all their local paths. Such a graph is called a *Local Causality Graph* (LCG), and abstracts all the executions of the AN.

##### **Definition 5**

The *Local Causality Graph* of an AN $(\Sigma ,S,T)$ is a tripartite digraph $(L,\mathcal O,P,E)$ where $L$, $\mathcal O$, $P$ are the vertices and *E* the edges such that:

$$\begin{aligned} L &\mathop{=}^{\Delta } \,\bigcup _{a\in \Sigma } S(a)\\ \mathcal O &\mathop{=}^{\Delta } \, \{ {{a}_{i}}\!\leadsto \!{{a}_{j}}\mid a\in \Sigma , a_i\in S(a), a_j\in S(a) \}\\ P&\mathop{=}^{\Delta } \, \bigcup _{{{a}_{i}}\!\leadsto \!{{a}_{j}}\in \mathcal O} \mathrm{local}\text{-}\mathrm{paths}({{a}_{i}}\!\leadsto \!{{a}_{j}})\\ E&\mathop{=}^{\Delta } \,\{ (a_j,{{a}_{i}}\!\leadsto \!{{a}_{j}}) \mid {{a}_{i}}\!\leadsto \!{{a}_{j}}\in \mathcal O\} \\ & \quad \cup \{ ({{a}_{i}}\!\leadsto \!{{a}_{j}},{\tau }) \mid {{a}_{i}}\!\leadsto \!{{a}_{j}}\in \mathcal O, {\tau }\in \mathrm{local}\text{-}\mathrm{paths}({{a}_{i}}\!\leadsto \!{{a}_{j}})\}\\ &\quad \cup \{ ({\tau },b_k) \mid {\tau }\in P, b_k\in {\mathrm{enab}({\tau })}\} \end{aligned}$$

From a complexity point of view, local paths are computed for each pair of local states within every automata. Since the length of a local path is at most the number of local states within the automaton, the number of local paths is at most polynomial in the number of local transitions and exponential in the size of the single automaton. In practice, the automata are small, typically between 2 and 4 states for biological models. Therefore, LCGs turn out to be very small compared to the reachable state space of biological networks. They have been successfully applied for analysing dynamics of ANs with hundreds or thousands of automata, which were intractable with standard model checking approaches [13, 17].

The over-approximation and under-approximation reduce to finding sub-graphs of LCGs that satisfy some particular structural properties, which have been proven to be necessary or sufficient for the reachability property, respectively. The over-approximation reduces here to finding an acyclic sub-graph that contains the main objective ${{g}_{0}}\!\leadsto \!{{g}_{1}}$ where leaves are empty local paths, and initial states match with the given initial state. This condition can be verified in a time linear with the LCG size [13]. The under-approximation we consider in the paper requires to find an acyclic sub-graph where all leaves are empty local states, where conditions of local paths (${\mathrm{enab}({\tau })}$) are independent, and which contain all possible objectives that can be involved for the goal reachability [14]. This requires enumerating over many possible sub-LCGs, but checking if a sub-LCG satisfies the sufficient condition is linear in its size, leading to an NP formulation.

##### **Theorem 1**

(Reachability over-approximation [13])* Given a state* $s\in S$, $g_1\in L$ *is reachable from* *s*,* i.e., there exists* $s'\in S$ *such that* $s\rightarrow ^{*}s'$, *only if* ${s({g})}\!\leadsto \!{g_1}\in \Omega$,* where* $\Omega \subseteq \mathcal O$ *is the least fixpoint of the monotonic function* $\mathrm{F}:2^{\mathcal O}\rightarrow 2^{\mathcal O}$ *with* $\mathrm{F}(\Omega ) \mathop {=}\limits ^{\Delta }\{ {{a}_{i}}\!\leadsto \!{{a}_{j}}\in \mathcal O\mid \exists {\tau }\in \mathrm{local}\text{-}\mathrm{paths}({{a}_{i}}\!\leadsto \!{{a}_{j}}): \forall b_k\in {\mathrm{enab}({\tau })}, {s({b})}\!\leadsto \!{b_k}\in \Omega \}.$

##### **Theorem 2**

(Reachability under-approximation [14])* Given a state* $s\in S$, $g_1\in L$ *is reachable from* *s*,* i.e., there exists* $s'\in S$ such that $s\rightarrow ^{*}s'$, *if there exists a sub-LCG* $(L',\mathcal O',P',E')$ *with* $L'\subseteq L$, $\mathcal O'\subseteq \mathcal O$, $P'\subseteq P$, $E'\subseteq E$,* such that*

- $$g_1\in L'$$;
- $$\forall a_j\in L'$$, $$(a_j,{s({a})}\!\leadsto \!{a_j})\in E'$$ *and* $$\forall a_i\in L',a_i\ne a_j$$, $$(a_j,{{a}_{i}}\!\leadsto \!{{a}_{j}})\in E'$$;
- $$\forall {{a}_{i}}\!\leadsto \!{{a}_{j}}\in \mathcal O'$$, $$\exists {\tau }\in \mathrm{local}\text{-}\mathrm{paths}({{a}_{i}}\!\leadsto \!{{a}_{j}}): ({{a}_{i}}\!\leadsto \!{{a}_{j}},{\tau })\in E'$$,
- $$\forall {\tau }\in P', \{ ({\tau },b_k) \in E\}\subseteq E'$$;

*and which verifies the following properties*:

- $$(L',\mathcal O',P',E')$$ *is acyclic*
- $\forall {\tau }\in P'$, $\forall n\in \{1,\dots ,|{\tau }|\}$,* there exists at most one* $a_i\in \mathrm{enab}({\tau }^n)$ *such that* $\forall b_j\in \mathrm{enab}({\tau }^n),b_j\ne a_i$, $S(a) \cap {\text {conn}}_{E'}(b_j) \nsubseteq \{a_i\}$.

*where* ${\text {conn}}_{E'}(v)$ *is the set of vertices connected to* *v*.

Figure 2 gives examples of sub-LCGs which approximate the reachability of $a_2$ in the AN of Fig. 1. The left LCG does not satisfy the necessary condition (no local paths from $c_2$ to $c_0$), hence $a_2$ is not reachable from the given initial state $\langle a_1,b_0,c_2 \rangle$. The middle LCG does satisfy the necessary condition. Finally, the right LCG is a valid sub-LCG for the sufficient condition for $a_2$ reachability. Whereas these examples show only acyclic LCGs, in general, cycles can exist in the causality analysis, revealing cyclic (non-solvable) dependencies between transitions.

### ASP syntax and semantics

Answer-Set Programming allows for automatic logical deductions thanks to an ASP model which declares variables, domains, and constraints, and to a solver which computes the solutions, possibly accounting for optimisation criteria. It is close to SAT (propositional satisfiability) [22] and known to be efficient for enumerating solutions of NP problems while providing a convenient language for specifying the model.

We give a very brief overview of ASP syntax and semantics that we use in the next section. Please refer to [10, 23, 24] for an in-depth introduction to ASP.

An ASP program is a Logic Program (LP) formed by a set of logical rules, composed of first order logic predicates, of the form:


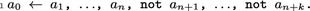
 where $a_i$ are (variable-free) atoms, i.e., elements of the Herbrand base, which is composed of all the possible predicates of the LP. The Herbrand base is built by instantiating the LP predicates with the LP terms (constants or elements of the Herbrand universe).

Essentially, such a logical rule states that when all $a_1,\dots ,a_n$ are true and all $a_{n+1},\dots ,a_{n+k}$ cannot be proven to be true, then $a_0$ has to be true as well. In the case where $a_0$ can be $\bot$ (and is omitted), the rule becomes: 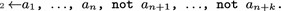 Such a rule is satisfied only if the right hand side of the rule is false (at least one of $a_1,\dots ,a_n$ is false or at least one of $a_{n+1},\dots ,a_{n+k}$ is true). On the other hand, a_0_ ← T ($a_0$ is always true) is abbreviated as a_0_. A solution (answer set) is a *stable* Herbrand model, that is, a minimal set of true atoms without variables (grounded atoms) where all the logical rules are satisfied.

ASP allows using variables (starting with an upper-case) instead of terms/predicates: these *pattern* declarations will be expanded to the corresponding propositional logic rules prior to the solving. For instance, the following ASP program has as unique (minimal) solution b(1) b(2) c(1) c(2). 
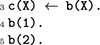
 In the following, we also use the notation *n* {a(X):b(X)} *m* which is satisfied when at least *n* and at most *m* a(X) are true where X ranges over the true b(X). This type of rule is usually used to generate solution candidates.

## Results

### Bifurcations

Given an initial state $s_0$ and a *goal* local state, a *bifurcation transition* is a transition from a state where the goal is reachable to a state where the goal is not reachable, i.e., there exists no sequence of transitions that leads to a state containing the goal local state. This implies that there exists at least one reachable attractor which does not contain a goal state.

Let us consider the AN of Fig. 1, with $s_0=\langle a_0,b_0,c_0 \rangle$ and the goal $a_2$. Figure 3 shows all the possible transitions from $s_0$.

The states with a grey background are connected to a state containing $a_2$ (in thick-blue). The transitions in thick-red are bifurcations: once in a white state, there exist no sequence of transitions leading to $a_2$. The white states constitute an attractor of the state graph from which it is not possible to reach a state containing $a_2$. In other words, bifurcations are the transitions from a grey state to a white state. Note that each transition between two global states is generated by one (and only one) local transition in the AN. In this example, $t_8$ is the (unique) local transition responsible for bifurcations from $s_0$ to $a_2$.

Given an AN $(\Sigma ,S,T)$, we search to identify the *local* transitions $t_b\in T$ which trigger a bifurcation from a state reached from $s_0\in S$ for a given goal, which describes a set of states $S_g\subseteq S$. We call $s_b$ a global state where a bifurcation occurs, and $s_u$ the global state after the bifurcation: $s_u = s_b\cdot t_b$. The goal is reachable from $s_b$ but not from $s_u$. This is illustrated by Fig. 4. Note that, as illustrated, $s_b$ is not inevitably reached: we allow the existence of alternative paths of transitions to the goal.

Definition 6 formalizes the notion of bifurcation, where the goal is specified by a local state $g_1$ (hence $S_g=\{ s\in S\mid s({g})=g_1\}$). Note that this goal specification does not loose generality, as one can build an automaton *g* with local states $g_0$ and $g_1$, and with a local transitions from $g_0$ to $g_1$ conditioned by each desired goal state.

#### **Definition 6**

(*Bifurcation transition*) Given an AN $(\Sigma ,S,T)$, a global state $s_0\in S$ and a goal local state $g_1$ with $g\in \Sigma$ and $g_1\in S(g)$, a *bifurcation transition* is a transition $s_b\xrightarrow {t_b} s_u$ of the AN with $s_b,s_u\in S$ and $t_b\in T$, such that (1) $s_0\rightarrow ^{*}s_b$; (2) $\exists s\in S$ where $s({g})=g_1$ with $s_b\rightarrow ^{*}s$; and (3) $\forall s'\in S$ where $s_u\rightarrow ^{*}s'$, $s'({g}) \ne g_1$.

Alongside the enumeration of candidate $s_b$ and $t_b$, reachability checking is at the core of the bifurcation identification.

Given a local transition $t\in T$ of an AN, the property of bifurcation transition for $g_1$ from initial state $s_0$ can be formulated in CTL [25] as:

1 $$\begin{aligned} s_0 \Rightarrow \mathsf {EF} \left( \mathrm{orig}(t)\wedge \mathrm{enab}(t) \wedge \mathsf {EF}~(g_1) \wedge \mathsf {EX} (\mathrm{dest}(t)\wedge \lnot \mathsf {EF}~(g_1)) \right) \end{aligned}$$

where $\mathsf {E}$ is the path existence operator, $\mathsf F$ the eventually operator, and $\mathsf X$ the next operator.

As explained in the introduction, verifying such a CTL property is a PSPACE-complete problem. In the rest of this paper, we introduce NP approximations of the bifurcation property that can be verified by a SAT/ASP solver.

### Identification of bifurcations using ASP

Among the states reachable from $s_0$, we want to find a state $s_b$ from which (1) the goal is reachable and (2) there exists a transition to a state from which the goal is not reachable. Putting aside the complexity of reachabilities checking, the enumeration of candidate states $s_b$ is a clear bottleneck for the identification of bifurcations in an AN.

Our approach combines the formal approximations and (optionally) unfoldings introduced in the previous section with a constraint programming approach to efficiently identify bifurcations. As discussed in the previous section, checking the over-/under-approximations from candidate states and sub-LCGs is easy. For the case of unfolding, checking if a state *s* belongs to the state space represented by a complete finite prefix is NP-complete [26]. Therefore, a declarative approach such as ASP [10] is very well suited for specifying admissible $s_b$ and $t_b$, and obtaining efficient enumerations of solutions by a solver.

We first present the general scheme of our method, and then given details on its implementation with ASP.

#### General scheme

A sound and complete characterization of the local transitions $t_b\in T$ triggering a bifurcation from state $s_0$ to the goal $g_1$ would be the following: $t_b$ is a bifurcation transition if and only if there exists a state $s_b\in S$ such that

$$\begin{aligned} {\mathrm{(C1)}}&s_u\not \rightarrow ^{*}g_1&{\mathrm{(C2)}}&s_b\rightarrow ^{*}g_1&{\mathrm{(C3)}}&s_0\rightarrow ^{*}s_b \end{aligned}$$

where $$s_u = s_b\cdot t_b$$, $$s_u \not \rightarrow ^{*}g_1 \mathop {\Leftrightarrow }\limits ^{\Delta }\forall s'\in S, s_u \rightarrow ^{*}s' \Rightarrow s'({g})\ne g_1$$ and $$s_b\rightarrow ^{*}g_1\mathop {\Leftrightarrow }\limits ^{\Delta }\exists s_g\in S: s_g({g}) = g_1\wedge s_b\rightarrow ^{*}s_g$$.

However, in an enumeration scheme for $s_b$ candidates, checking reachability and non-reachability of the goal from each $s_b$ candidate ((C1) and (C2)) is prohibitive. Instead, we relax the above constraints as follows:

$$\begin{aligned} {(\mathrm{I1}^\#)}&\,\lnot \mathrm{OA}(s_u\rightarrow ^{*}g_1)&{(\mathrm{I2}^\#)}&\,\mathrm{UA}(s_b\rightarrow ^{*}g_1)&\begin{array}{ll} ({\mathrm{I3}})&{}s_b\in \mathrm{unf}\text{-}\mathrm{prefix}(s_0)\\ ({\mathrm{I3}^\#})&{}\mathrm{UA}(s_0\rightarrow ^{*}s_b) \end{array} \end{aligned}$$

where $\mathrm{unf}\text{-}\mathrm{prefix}(s_0)$ is the set of all reachable states from $s_0$ represented as the prefix ofcomputed (see “Background” and “State graph and partial order reductions”). Either $(\mathrm{I3})$ or $(\mathrm{I3}^\#)$ can be used, at discretion. Recall that $\mathrm{UA}(s\rightarrow ^{*}s')\Rightarrow s\rightarrow ^{*}s' \Rightarrow \mathrm{OA}(s\rightarrow ^{*}s')$ [13, 14] (see “Background”/“Formal approximations”), thus we obtain the following implications:

$$\begin{aligned} ({\mathrm{I1}^\#})&\Rightarrow {(\mathrm{C1})}&({\mathrm{I2}^\#})&\Rightarrow ({\mathrm{C2}})&\begin{array}{ll} ({\mathrm{I3}})&{}\Leftrightarrow ({\mathrm{C3}})\\ ({\mathrm{I3}^\#})&{}\Rightarrow ({\mathrm{C3}}) \end{array} \end{aligned}$$

Therefore, our characterization is sound (no false positive) but incomplete: some $t_b$ might be missed (false negatives). Using $(\mathrm{I3})$ instead of $(\mathrm{I3}^\#)$ potentially reduces the false negatives, at the condition that the prefix of the unfolding is tractable. When facing a model too large for the unfolding approach, we should rely on $(\mathrm{I3}^\#)$ which is much more scalable but may lead to more false negatives.

Relying on the unfolding from $s_b$ ($\mathrm{unf}\text{-}\mathrm{prefix}(s_b)$) is not considered here, as it would require to compute a prefix from each $s_b$ candidate, whereas $\mathrm{unf}\text{-}\mathrm{prefix}(s_0)$ is computed only once before the bifurcation identification.

#### Complexity

The decision of $(\mathrm{I1}^\#)$, $(\mathrm{I2}^\#)$, and $(\mathrm{I3}^\#)$ can be formulated as NP problems in the size of the LCG. Recall that the size of the LCG is polynomial with the number of local states and local transitions in the AN, and exponential with the number of local states within a single automaton.

The decision of $(\mathrm{I3})$ is NP-complete with respect to the size of the prefix of the unfolding, which computation is PSPACE [12]. Nevertheless, checking if $(\mathrm{I1}^\#)$, $(\mathrm{I2}^\#)$, and $(\mathrm{I3})$ are satisfied can remain more tractable than checking the exact CTL property: $(\mathrm{I3})$ uses the (complete) set of reachable states, but does not require the transitions.

#### ASP implementation

We present here the main rules for implementing the identification of bifurcation transitions with ASP. A significant part of ASP declarations used by $(\mathrm{I1}^\#)$, $(\mathrm{I2}^\#)$, $(\mathrm{I3})$, and $(\mathrm{I3}^\#)$ are generated from the prior computation of $\mathrm{local}\text{-}\mathrm{paths}$ and, in the case of $(\mathrm{I3})$, of the prefix of the unfolding. Applied on Fig. 1, our implementation correctly uncovers $t_8$ as a bifurcation for $a_2$.

*Problem instance: local states, transitions, and states* Every local state $a_i\in S(a)$ of each automaton $a\in \Sigma$ is declared with the predicate 1s(*a*, *i*). We declare the local transitions of the AN and their associated conditions by the predicates tr(*id*,* a*,* i*,* j*) and trcond(*id*,* b*,* k*), which correspond to the local transition ${a}_{i} \xrightarrow {\{b_k\}\cup \ell } {a}_{j}\in T$. States are declared with the predicate s(ID, A, I) where ID is the state identifier, and A, I, the automaton and local state present in that state. Finally, the goal $g_1$ is declared with goal(*g*, 1).

For instance, the following instructions declare the automaton *a* of Fig. 1 with its local transitions, the state $s_0=\langle a_0,b_0,c_0 \rangle$, and the goal being $a_2$: 
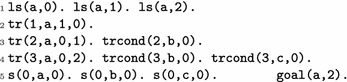


*Solution candidates* $t_b$ *and associated definitions for* $s_b$ *and* $s_u$ The bifurcation transition $t_b$, declared as btr(*b*), is selected among the declared transitions identifiers (line 6). If ${a}_{i} \xrightarrow {\ell } {a}_{j}$ is the selected transition, the global state $s_u$ (recall that $s_u=s_b\cdot t_b$) should satisfy $s_u({a}) = a_j$ (line 7) and, $\forall b_k\in \ell$, $s_u({b}) = b_k$ (line 8). The state $s_b$ should then match $s_u$, except for the automaton *a*, as $s_b({a})=a_i$ (lines 9, 10). 
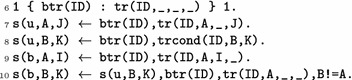


$(\mathrm{I1}^\#)$ *Integrity constraint to verify* $\lnot \mathrm{OA}(s_u\rightarrow ^{*}g_1)$ This part aims at imposing that the defined state $s_u$, for a candidate bifurcation transition $t_b$ (lines 7 and 8), should not reach $g_1$. For that, we designed an ASP implementation of the reachability over-approximation presented in “Background” section (“Formal approximations”). It consists in building a Local Causality Graph (LCG) from pre-computed $\mathrm{local}\text{-}\mathrm{paths}$ oa_valid(G, 1s(A, I)). A predicate is then defined upon the over-approximation LCG *G* to be true when the local state $a_i$ is reachable from the initial state $s_G$. The full implementation is given in Additional file 1. Therefore, in order to ensure that the defined state $s_u$ does not reach the goal $g_1$, we forbid the fact that there exists an LCG, built from the initial state $s_u$, that contains a local state $g_1$, where $g_1$ is the goal of the problem, associated to the predicate $\texttt {goal}$ (line 11).
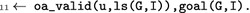


$(\mathrm{I2}^\#)$ *Verification of* $\mathrm{UA}(s_b\rightarrow ^{*}g_1)$ This part aims at imposing that the defined state $s_b$, for a candidate bifurcation transition $t_b$, (lines 7 and 8) should reach $g_1$. Our designed ASP implementation of the reachability under-approximation consists in finding a sub-LCG *G* with the satisfying properties for proving the sufficient condition. If such a graph exists, then the related reachability property is true. The edges of this sub-LCG are declared with the predicate ua_1cg(G, Parent, Child). The graph is parameterized by (1) a *context* which specifies a set of possible initial states for the objectives and (2) an edge from the node root to the local state(s) for which the simultaneous reachability has to be decided. The full implementation is given in Additional file 1. We instantiate the under-approximation LCG for building a state $s_b$ from which the goal $g_1$ is reachable by imposing the following rules. First, $g_1$ is a child of the root node of graph *b* (line 12). Second, the context is subject to the same constraints as $s_b$ from $s_u$ (lines 13 and 14 reflect lines 9, and 10). Then, $s_b$ defines one local state per automaton among the context from which the reachability of $g_1$ is ensured (line 15), and according to lines 9, and 10. The rules in lines 12, 13, 14, and 15 will be the entry point for building an under-approximation LCG, and rules imposed in Additional file 1 will allow to further develop the LCG from these entry points. If the integrity constraints in Additional file 1 reject the provided entry points, then the reachability from $s_b$ to $g_1$ does not hold. Thus, the defined $s_b$ is not valid and the selected $t_b$ will not be an answer set of the program.
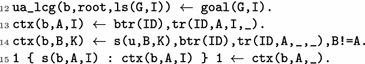


$(\mathrm{I3})$ *Verification* of $s_b\in \mathrm{unf}\text{-}\mathrm{prefix}(s_0)$ Given a prefix of an unfolding from $s_0$ , checking if $s_b$ is reachable from $s_0$ is an NP-complete problem [26] which can be efficiently encoded in SAT [27] (and hence in ASP). A synthetic description of the ASP implementation of reachability in unfoldings is given in Additional file 1. The interested reader should refer to [21]. Our encoding provides a predicate reach(*a*,* i*) which is true if a reachable state contains $a_i$. Declaring $s_b$ reachable from $s_0$ is done simply as follows: 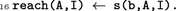


*(* $\mathit{I3}^\#$ *)* *Verification* of $\mathrm{UA}(s_0\rightarrow ^{*}s_b)$ An alternative to $(\mathrm{I3})$ which does not require to compute a complete prefix of the unfolding is to rely on the under-approximation of reachability similarly to $(\mathrm{I2}^\#)$. The under-approximation is instantiated for the reachability of $s_b$ from $s_0$ with the following statements: 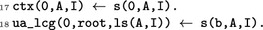


## Experiments

We evaluated our method in three real biological networks case studies that show differentiation capabilities. We selected networks that show at least two attractors reachable from the same initial state. For each network, we supplied a goal state representing one attractor. Thus, the goal state is a state reachable from the selected initial state. Because at least one attractor is reachable from the same selected initial state, transitions that lead to the other attractors are by definition bifurcation transitions. We aimed at identifying transitions that cause a bifurcation for the reachability of the goal state. The three case studies used are briefly described in the following paragraphs.

### Models, initial states, and goals

#### Immunity control in bacteriophage lambda (*Lambda phage*)

In temperate bacteriophages the choice of entering lysis and lysogenization cycles is controlled by bacterial and viral genes. In the lambda case, at least five viral genes (refered to as cI, cro, cII, N and cIII) and several bacterial genes were identified. We applied our method on an AN equivalent to the model introduced in [28]. Based on this study we selected one initial state and two different goals, corresponding to lysis or lysogenization phases both reachable from the initial state. The lysis phase is characterized by the attractor $\{\langle CI_0, Cro_2, CII_0, N_0 \rangle , \langle CI_0, Cro_3, CII_0, N_0 \rangle \}$, while the lysogenization phase, by $\{\langle CI_2, Cro_0, CII_0, N_0 \rangle , \langle CI_2, Cro_0, CII_1, N_0 \rangle \}$. The initial state was $\langle CI_0, Cro_0, CII_0, N_0 \rangle$. The selected goals where $CI_2$ (lysogenization attractor) and $Cro_2$ (lysis attractor). One can not access the lysogenization goal from the lysis attractor and vice versa.

#### Epidermal growth factor and tumor necrosis factor$_{\alpha }$

EGF/TNF is a model that combines two important mammalian signaling pathways induced by the epidermal growth factor (EGF) and tumor necrosis factor alpha (TNF$_{\alpha }$) [29, 30]. EGF and TNF$_{\alpha }$ ligands stimulate ERK, JNK and p38 MAPK cascades, the PI3K/AKT pathways, and the NFkB cascade. This network of 28 components encompasses cross-talks between these pathways as well as two negative feedback loops. We applied our method from the initial state corresponding to the signal TNF$_\alpha$ active and EGF inactive; the two goals refer to downstream proteins, namely the inactivation of NBkB and the activation of its inhibitor, IKB.

#### T-helper cell plasticity

T-helper cell has been studied in [8] in order to investigate switches between attractors subsequent to changes of input conditions. It is a cellular network regulating the differentiation of T-helper (Th) cells, which orchestrate many physiological and pathological immune responses. T-helper (CD4+) lymphocytes play a key role in the regulation of the immune response. By APC activation, native CD4 T cells differentiate into specific Th subtypes producing different cytokines which influence the activity of immune effector cell types. Differentiation in one subtype rather than another depends on the presence of specific polarizing cytokine combinations. These different lineages are characterized by a set of cytokines they express under the control of a *master regulator* transcriptional factor. Each master regulator is critically involved in the driving of the differentiation of the Th lineage they specify. The network is composed of 101 nodes and 221 interactions; the corresponding AN has in total 381 local transitions. Note that due to the very high number of reachable states from some particular initial states of the network, the authors in [8] had to analyse a reduced version of this network, which does not preserve all the reachability properties. In this work, we analyse the full model. We selected initial states and goals for this model according to the attractors identified in [8].

We applied our method for three different initial states, namely th1, th2, and pluri. The two formers are arbitrary initial states from which particular subtypes (Th1 and Th2, respectively) are reachable. The “pluri” initial state corresponds to a potential cell environment which can trigger a differentiation among different cell subtypes (the differentiation is non-deterministic in the Boolean model): the initial states specify that APC, IL1B$_e$, IL25$_e$, IL27$_e$, IL29$_e$, IL2$_e$, IL33$_e$, IL36$_e$, IL4$_e$, and TGFB$_e$ ($_e$ stands for environment) are active, and only them.

In all cases, the goals correspond to the activation of master regulators and cytokines which are specific markers for differentiated Th subtypes.

### Methods

Given an AN, an initial state, and a goal, we performed the bifurcation identification with three different methods:

1. Exact model checking using NuSMV [20]: for each local transition in the AN specification, we verify if it is a bifurcation transition according to the CTL formula given in Eq. 1. This identification is exact and complete, but has a high theoretical complexity.
2. ASP solving of $(\mathrm{I1}^\#)$, $(\mathrm{I2}^\#)$, and $(\mathrm{I3})$ (computation of the reachable states set from $s_0$). We use clingo 4.5.3 [31] as ASP solver, and Mole [32] for the computation of the complete finite prefix for $(\mathrm{I3})$. This identification is exact but incomplete: some bifurcation transitions can be missed.
3. ASP solving of $(\mathrm{I1}^\#)$, $(\mathrm{I2}^\#)$, and $(\mathrm{I3}^\#)$ (reachability under-approximation). We use clingo 4.5.3 [31] as ASP solver. This identification is exact but incomplete: some bifurcation transitions can be missed. Due to the additional approximations brought by $(\mathrm{I3}^\#)$ compared to $(\mathrm{I3})$, it is expected that less bifurcation transitions can be identified with this latter approach, but with a higher scalability.

The computation times correspond to the total toolchain duration, and includes the $\mathrm{local}\text{-}\mathrm{paths}$ computation, unfolding, ASP program generation, ASP program loading and grounding, and solving. Note that the LCG (see above “Background” and “Results” sections) computation (and ASP program generation) is almost instantaneous for each case. We implemented the three methods in the Pint software.^2^ Models and instructions are provided in Additional file 2.

### Results

Table 1 summarizes the results of the identification of bifurcation transition for the models, initial states and goals described above. In the remainder of this section, we discuss two aspects of these results: the scalability of our approach and the biological interpretation of the identified bifurcations.

#### Scalability

For the analysed models, exact model checking and approximation using $(\mathrm{I3})$ give comparable execution times, with nevertheless an advantage for $(\mathrm{I3})$ in most cases. Because the model checking approach is exact, the identified bifurcation transitions is complete, whereas, due to $(\mathrm{I1}^\#)$ and $(\mathrm{I2}^\#)$ approximations, the second approach generally identifies less bifurcation transitions. As supported by the experiments on Th_th2, the computation of $(\mathrm{I3})$ should be, in practice, more tractable than the verification of the CTL expression of Eq. 1. Indeed, $(\mathrm{I3})$ requires only to compute the *set* of reachable states, where CTL verification requires, in addition, to store the transitions between these states.

Importantly, both methods fail on the Th_pluri model (no result after 2 h). This can be explained by the very large reachable dynamics. In the case of model checking, we emphasize that NuSMV fails due to the size of the model, and it has been able to verify none of the supplied CTL properties. In the case of $(\mathrm{I3})$, the failure is due to the complete finite prefix computation which does not terminate in due time; this suggests that the reduction relying on concurrent transitions is not sufficient for this particular model to achieve a tractable representation of the reachable state space. Future work may consider other symbolic representations of the reachable state space, notably using BDDs and variants [19].

The third approach, using the additional approximation $(\mathrm{I3}^\#)$ is tractable on the large model, supporting a higher scalability of this latter approach. Indeed, the computation of the finite complete prefix for $(\mathrm{I3})$ is PSPACE-complete, solving $(\mathrm{I3}^\#)$ is NP (with LCG size). Whereas, the difference between PSPACE and NP complexity classes is not known, it is a common observation *in practice* that NP solving (notably using SAT) is more tractable than PSPACE solving. As expected, in the smaller models, less bifurcation transitions than the former approaches are returned. Concerning the ASP grounding and solving computation times (data not shown) the grounding time depends on the model size and is independent of the choice of the initial state and goal; whereas in the case of the solving time, the choice of the initial state may have an important impact. This effect appears much more visible in the larger T-helper model. Grounding time has very small and similar values ($\approx$0.05s) for the small and middle size models (4–22 automata and 11–55 transitions). However in the larger model (six times more transitions) the grounding time raises to 2 orders of magnitude. Solving time behaves differently, while it remains small and similar for small and middle size models. It raises to 4 orders of magnitude in the case of the larger model. Across all studied models the proportion of grounding and solving time against total computation time varies from 14–61% for grounding and 19–71% for solving. We observe that in the small and middle size models the grounding and solving proportion remains quite similar, while the grounding time proportion is much smaller than the solving one in the large-scale model.

#### Biological interpretation

We illustrate here how bifurcation transitions should be interpreted with the example of Th_pluri model for bifurcations from FOXP3 active. The four identified bifurcation transitions are the following:

- STAT6 0 $\rightarrow$ 1 when IL4R=1
- RORGT 0 $\rightarrow$ 1 when BCL6=0 and FOXP3=0 and STAT3=1 and TGFBR=1
- STAT1 0 $\rightarrow$ 1 when IL27R=1
- STAT1 0 $\rightarrow$ 1 when IFNGR=1

These transitions are local transitions of the AN which satisfy $(\mathrm{I1}^\#)$, $(\mathrm{I2}^\#)$, and $(\mathrm{I3}^\#)$. The first transition corresponds to the activation of STAT6 by IL4R, the second is the joint activation of RORGT by STAT3 and TGFBR provided that BCL6 and FOXP3 are inactive, and the third and fourth are the activation of STAT1 either by active IL27R or by active IFNGR.

The fact that these transitions are bifurcation transitions for FOXP3 means the following: starting from the specified initial state, there exists future states where the occurence of one of these transitions puts the system in a state where FOXP3 is no longer activable, and in particular, all future attractors have FOXP3 inactive. In that precise case, the active form of FOXP3 is a marker for the “Treg” Th subtype: hence, these 4 bifurcation transitions can prevent the differentiation of the cell in this type.

## Conclusions

This paper presents an original combination of computational techniques to identify transitions of a dynamical system that can remove its capability to reach a (set of) states of interest. Our methodology combines static analysis of ANs dynamics, partial order representations of the state space, and constraint programming to efficiently enumerate those bifurcations. To our knowledge, this is the first integrated approach for deriving bifurcation transitions from concurrent models, and ANs in particular.

Bifurcations are key features of biological networks, as they model decisive transitions which control the *differentiation* of the cell: the bifurcations decide the portions of the state space (no longer) reachable in the long-run dynamics. Providing automatic methods for capturing those differentiations steps is of great interest for biological challenges such as cell reprogramming [8, 33], as they suggest targets for modulating undergoing cellular processes. Our approach is focused on non-deterministic discrete dynamics, in opposition to deterministic systems, such as piecewise-affine systems on which differentiation is determined by the initial state in a continuous space [34].

Bifurcation transitions can be modelled as CTL properties and verified by exploring the reachable state and transition space. Our method aims at circumventing the state space explosion problem for large networks thanks to the formal approximations of reachability properties.

Given an initial state of the AN and a goal state, our method first computes static abstractions of the AN dynamics and (optionally) a symbolic representation of the reachable state space with so-called unfoldings. From those prior computations, a set of constraints is issued to identify bifurcation transitions. We used ASP to declare the admissible solutions and the solver clingo to obtain their efficient enumerations. For large models, the unfolding may be intractable: in such a case, the methods relies only on reachability over- and under-approximations. By relying on those relaxations which can be efficiently encoded in ASP, our approach avoids costly exact checking, and is tractable on large models, as supported by the experiments.

For applications when the initial state is not fully determined, or equivalently, a set of initial states has to be considered, our approach, including CTL and approximations, can be easily extended for the identification of *universal* bifurcation transitions: such transitions are bifurcation transitions for every candidate initial state. Indeed, the verification of CTL properties is universal, as well as the implemented under-approximation of reachability $(\mathrm{I3}^\#)$. The unfolding prefix $(\mathrm{I3})$ can also be extended to multiple initial states [11]. The identification of *existential* bifurcation transitions, i.e., such that there exists at least one candidate initial state for which the transition is a bifurcation transition, could also be implemented for the approximation $(\mathrm{I3}^\#)$ using ASP, but with a potential lower scalability.

Further work will consider the complete identification of bifurcation transitions, by allowing false positives (but no false negatives). In combination with the under-approximation of the bifurcations presented in this paper, it will provide an efficient way to delineate *all* the transitions that control the reachability of the goal attractor. Moreover, we will investigate the implementation of refined over- and under-approximations of reachability described in [13] for better capturing transition ordering constraints. Future work will also focus on exploiting the identified bifurcations for driving estimations of the probability of reaching the goal at steady state, in the scope of hybrid models of biological networks [35, 36].

## Additional files

**Additional file 1.** Details on ASP implementation. Providing a commented implementation in ASP for the identification of bifurcations using formal approximations.

**Additional file 2.** Models and scripts. Archive containing the model and scripts used for the experiments. Available at https://www.lri.fr/~pauleve/additional_file_2.zip.

## Footnotes

1
https://github.com/colomoto/bioLQM.

2
http://loicpauleve.name/pint.
