# Supplementary material for: Identification of bifurcation transitions in biological regulatory networks using Answer-Set Programming
Source: Algorithms Mol Biol. 2017 Jul 20;12:19. doi: 10.1186/s13015-017-0110-3 (PMC5520421; doi:10.1186/s13015-017-0110-3)
Supplement: Supplementary file 1 — Additional file 1. Details on ASP implementation. Providing a commented implementation in ASP for the identification of bifurcations using formal approximations. [file 13015_2017_110_MOESM1_ESM.pdf]

Additional file 1 — Details on ASP implementation

Louis Fippo Fitime, Olivier Roux, Carito Guziolowski, and Loïc Paulevé

Supplementary material for the article

A Over- and Under-approximation of Reachability

A.1 $\mathsf{OA}(s \to^* s')$: necessary condition for reachability

We propose here a possible encoding of the necessary condition for reachability in ANs outlined in the article and introduced in [1]. Starting from $s_u(g) = g_0$, the analysis starts with the *local paths* of the *objective* $g_0 \rightsquigarrow g_1$: g_1 is reachable only if all the conditions of the transitions of at least one local path $\eta \in \mathsf{local\text{-}paths}(g_0 \rightsquigarrow g_1)$ are reachable. This recursive reasoning can be modelled with a graph relating dependencies between objectives, local paths, and local states.

The local paths computed *a priori* are used to generate the template declaration of the directed edges of the LCG `oa_lcg(G,Parent,Child)` from each possible objective $a_i \rightsquigarrow a_j$. If $\mathsf{local\text{-}paths}(a_i \rightsquigarrow a_j) = \emptyset$, the objective $a_i \rightsquigarrow a_j$ is linked to a node `bottom`:

```
1 oa_lcg(G,obj(a,i,j),bottom) ← oa_lcg(G,_,obj(a,i,j)).
```

otherwise, for $\mathsf{local\text{-}paths}(a_i \rightsquigarrow a_j) = \{\eta^1, \dots, \eta^n\}$, we declare a node `lpath` for each different local path $m \in \{1, \dots, n\}$ as a child of $a_i \rightsquigarrow a_j$:

```
2 oa_lcg(G,obj(a,i,j),lpath(obj(a,i,j),m)) ← oa_lcg(G,_,obj(a,i,j)).
```

then, for each different local state $b_k \in \widetilde{\eta^m}$ in the conditions of the local transitions of η^m, we add an edge from the `lpath` node to `ls(b,k)`:

```
3 oa_lcg(G,lpath(obj(a,i,j),m),ls(b,k)) ← oa_lcg(G,_,obj(a,i,j)).
```

In the case when the local path requires no condition ($\widetilde{\eta^m} = \emptyset$, this can happen when the objective is trivial, i.e., $a_i \rightsquigarrow a_i$, or when the local transitions do not dependent on the other automata), we link the `lpath` to a node `top`:

```
4 oa_lcg(G,lpath(obj(a,i,j),m),top) ← oa_lcg(G,_,obj(a,i,j)).
```

A LCG G for over-approximation is parameterized with a state s_G: if a local path has a local state a_j in its transition conditions, the node `ls(a,j)` is linked, in G, to the node for the objective $a_i \rightsquigarrow a_j$ (line 5), with $a_i = s_G(a)$. It is therefore required that state s_G defines a (single) local state for each automaton referenced in G (line 6).

```
5 oa_lcg(G,ls(A,I),obj(A,J,I)) ← oa_lcg(G,_,ls(A,I)), s(G,A,J).
6 1 { s(G,A,J) : ls(A,J) } 1 ← oa_lcg(G, _, ls(A, _)).
```

The necessary condition for reachability is then declared using the predicate `oa_valid(G,N)` which is true if the node `N` satisfies the following condition: it is not `bottom` (line 7); and, in the case of a local state or objective node, one of its children is `oa_valid` (lines 8 and 9; or in the case of a local path, either `top` is its child, or all its children (local states) are `oa_valid` (lines 10 and 11).

```
7 ← oa_valid(G,bottom).
8 oa_valid(G,ls(A,I)) ← oa_lcg(G,ls(A,I),X),oa_valid(G,X).
9 oa_valid(G,obj(A,I,J)) ← oa_lcg(G,obj(A,I,J),X),oa_valid(G,X).
10 oa_valid(G,N) ← oa_lcg(G,N,top).
11 oa_valid(G,lpath(obj(a,i,j),m)) ← ⋀_{b_k ∈ η̃^m} oa_valid(G,ls(b,k)).
```

A.2 $\mathsf{UA}(s \to^* s')$: sufficient condition for reachability

We give here a declarative implementation of the sufficient condition for reachability in ANs outlined in the paper and introduced in [2]. The under-approximation consists in building a graph relating objectives, local paths, and local states which satisfies several constraints. If such a graph exists, then the related reachability property is true. Similarly to (I1$^\#$), we give template declarations for the edges with the predicate `ua_lcg(G,Parent,Child)`. We assume that the reachability property is specified by adding an edge from `root` to `ls(a,i)` for each local state to reach.

The graph `ua_lcg` is parameterized with a *context* which is a set of local states, declared with the predicate `ctx(G,A,J)`. Every local states a_i of the graph that are not part of the reachability specification belong to that context (line 12); and are linked to the objective $a_j \rightsquigarrow a_i$ for each a_j in the context (line 13).

```
12 ctx(G,A,I) ← ua_lcg(G,N,ls(A,I)), N != root.
13 ua_lcg(G,ls(A,I),obj(A,J,I)) ← ua_lcg(G,_,ls(A,I)), ctx(G,A,J).
```

A first constraint is that each objective in the graph is linked to one and only one of its local path. Therefore, objectives without local paths ($\mathsf{local\text{-}paths}(a_i \rightsquigarrow a_j) = \emptyset$) cannot be included (line 14), for the others, a choice has to be made among $\mathsf{local\text{-}paths}(a_i \rightsquigarrow a_j) = \{\eta^1, \dots, \eta^n\}$ (line 15).

```
14 ← ua_lcg(G,_,obj(a,i,j)).
15 1 { ua_lcg(G,obj(a,i,j),lpath(obj(a,i,j),1..n)) } 1 ← ua_lcg(G,_,obj(a,i,j)).
```

As for `oa_lcg`, each local path is linked to all the local states composing its transition conditions: for each $m \in \{1, \dots, n\}$, for each $b_k \in \widetilde{\eta^m}$,

```
16 ua_lcg(G,lpath(obj(a,i,j),m),ls(b,k)) ← ua_lcg(G,_,obj(a,i,j)).
```

The graph has to be acyclic. This is declared using a predicate `conn(G,X,Y)` which is true if the node `X` is connected (there is a directed path) to `Y` (line 17). A graph is cyclic when `conn(G,X,X)` (line 18).

```
17 conn(G,X,Y) ← ua_lcg(G,X,Y).  conn(G,X,Y) ← ua_lcg(G,X,Z), conn(G,Z,Y).
18 ← conn(G,X,X).
```

Then, if the node for an objective $a_i \rightsquigarrow a_j$ is connected to a local state a_k, the under-approximation requires $a_i \rightsquigarrow a_j$ to be connected with $a_k \rightsquigarrow a_j$ (assuming that a has at least 3 local states, definition not shown):

```
19 ua_lcg(G,obj(A,I,J),obj(A,K,J)) ← not boolean(A), conn(G,obj(A,I,J),ls(A,K)).
```

When a local transition is conditioned by at least two other automata (for instance $c_\circ \xrightarrow{a_i,b_j} c_\bullet$), the under-approximation requests that reaching b_j does not involve other local states from a others that a_i. This is stated by the `indep(G,Y,a,i,ls(b,j))` which cannot be true if b_j is connected to a local state a_k with $k \neq i$ line 20. Then, the under-approximation requires that at most one `indep` predicate is false, for a given LCG G and a given local path Y (line 21). Such an independence should also hold between the local states of the reachability specification (line 22).

```
20 indepfailure(Y,ls(A,I)) ← indep(G,Y,A,I,N), conn(G,N,ls(A,K)), K!=I.
21 ← indepfailure(Y,N),indepfailure(Y,M),M!=N.
22 indep(G,root,A,I,ls(B,J)) ← ua_lcg(G,root,ls(A,I)),ua_lcg(G,root,ls(B,J)),B != A.
```

For $\eta^m \in \mathsf{local\text{-}paths}(a_i \rightsquigarrow a_j)$, for each local transition $a_\circ \xrightarrow{\ell} a_\bullet \in \eta^m$, for each couple of different local states in its condition $b_k, c_l \in \ell, b_k \neq c_l$:

```
23 indep(G,lpath(obj(a,i,j),m),b,k,ls(c,l)) ← ua_lcg(G,_,lpath(obj(a,i,j),m)).
```

B Reachability in unfoldings

A (prefix of an) unfolding is an acyclic bipartite digraph where nodes are either *events* (application of a transition) or *conditions* (change of local state) [3]. We use the predicate `post(X,Y)` to denote an edge from X to Y; and `h(C,ls(A,I))` to denote that the condition C corresponds to the local state a_i. Figure 1 shows an example of unfolding.

A state *s* belongs to the prefix if it is possible to build a *configuration* such that all the local states in *s* have a unique corresponding condition on the *cut* of the configuration (line 1).

A configuration is a set of events, and we use `e(E)` to denote that the event E belongs to the configuration. By definition, if E is in a configuration, all its parent events are in the configuration (line 2). There should be no *conflicts* between two events of a configuration: two events are in conflict if they share a common parent condition (line 3).

A condition is on the cut if its parent event is in the configuration (line 4), and none of its children event is in the configuration (line 5).

```
1 1 { cut(C)  : h(C,ls(A,I)) } 1 ← reach(A,I).
2 e(F) ← post(F,C),post(C,E),e(E).
3 ← post(C,E),post(C,F),e(E),e(F),E != F.
4 e(E) ← cut(C),post(E,C).
5 ← cut(C),post(C,E),e(E).
```

References

[1] Paulevé, L., Magnin, M., Roux, O.: Static analysis of biological regulatory networks dynamics using abstract interpretation. Mathematical Structures in Computer Science **22**(04), 651–685 (2012)

[2] Folschette, M., Paulevé, L., Magnin, M., Roux, O.: Sufficient conditions for reachability in automata networks with priorities. Theoretical Computer Science **608 Part 1**, 66–83 (2015). From Computer Science to Biology and Back

[3] Esparza, J., Heljanko, K.: Unfoldings – A Partial-Order Approach to Model Checking. Springer, Berlin, Heidelberg (2008)

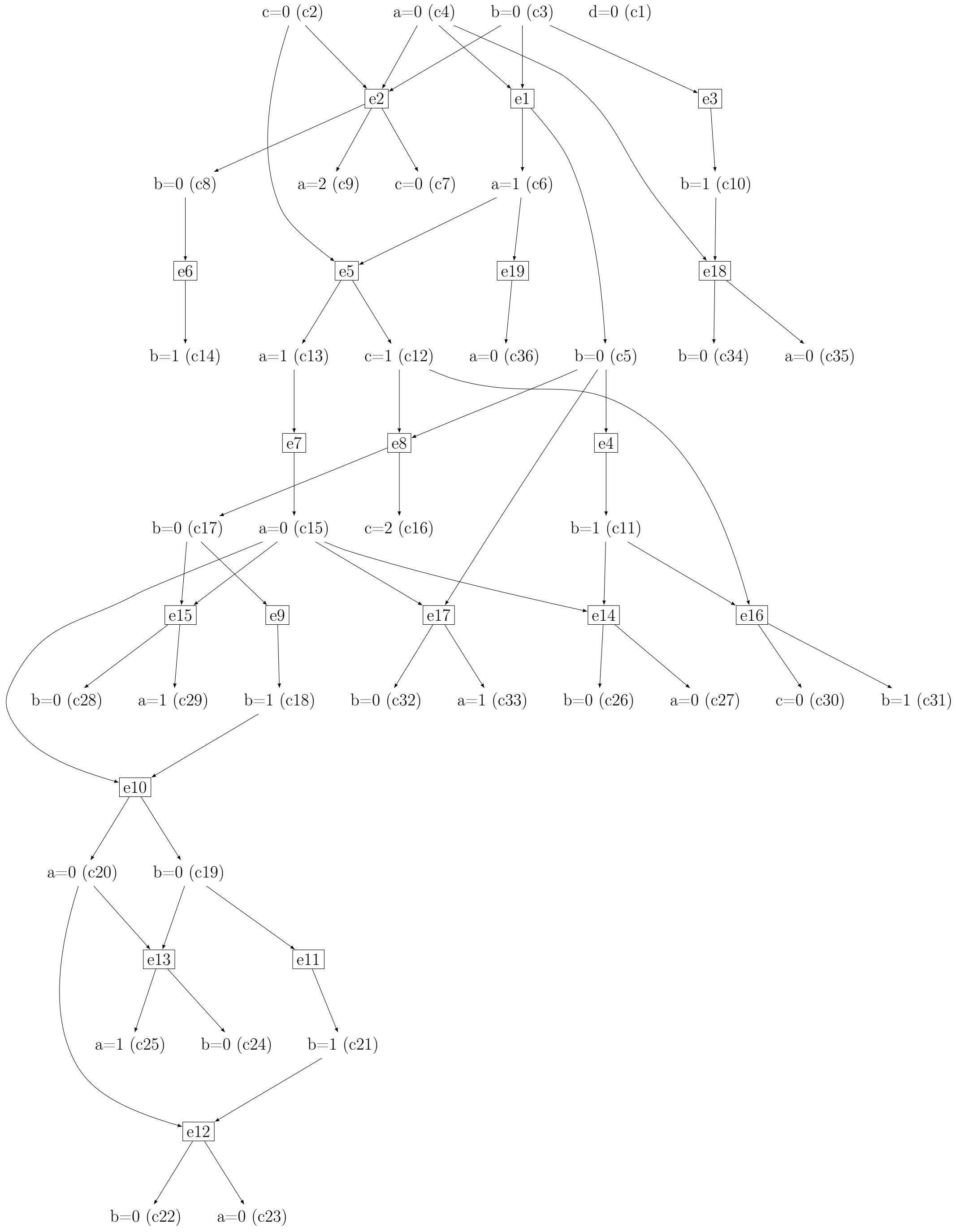

Figure 1: Unfolding of the AN of Figure 1 from the main article. Events are boxed nodes, conditions have no borders and indicate both the automata local state and the condition identifier.
